# The impact of geography and climate on the population structure and local adaptation in a wild bee

**DOI:** 10.1111/eva.13558

**Published:** 2023-05-08

**Authors:** Farida Samad‐zada, Evan P. Kelemen, Sandra M. Rehan

**Affiliations:** ^1^ Department of Biology York University Toronto Canada

**Keywords:** gene–environment associations, pollinators, population genomics, signatures of selection

## Abstract

Deciphering processes that contribute to genetic differentiation and divergent selection of natural populations is useful for evaluating the adaptive potential and resilience of organisms faced with various anthropogenic stressors. Insect pollinator species, including wild bees, provide critical ecosystem services but are highly susceptible to biodiversity declines. Here, we use population genomics to infer the genetic structure and test for evidence of local adaptation in an economically important native pollinator, the small carpenter bee (*Ceratina calcarata*)*.* Using genome‐wide SNP data (*n* = 8302), collected from specimens across the species' entire distribution, we evaluated population differentiation and genetic diversity and identified putative signatures of selection in the context of geographic and environmental variation. Results of the analyses of principal component and Bayesian clustering were concordant with the presence of two to three genetic clusters, associated with landscape features and inferred phylogeography of the species. All populations examined in our study demonstrated a heterozygote deficit, along with significant levels of inbreeding. We identified 250 robust outlier SNPs, corresponding to 85 annotated genes with known functional relevance to thermoregulation, photoperiod, and responses to various abiotic and biotic stressors. Taken together, these data provide evidence for local adaptation in a wild bee and highlight genetic responses of native pollinators to landscape and climate features.

## 1 INTRODUCTION

Evolutionary processes in wild populations are determined by a variety of factors, at different spatial and temporal scales (Chevin et al., 2010). Understanding the adaptive potential of a species requires considering both neutral and selective forces that have historically shaped the population of interest (Holderegger et al., 2006). Characterizing population structure, diversity, and gene flow is crucial for conservation as this helps identify biogeographical features that have shaped population dynamics over time (Hedrick, 2001; Manel et al., 2003). Another important goal of conservation genomics is elucidating the complex interactions between ecological processes and selection pressures (Otto, 2018). There is increasing interest in understanding how historical demography shaped by environmental and geographic variation affects population structure of species of conservation concern. Determining how natural populations have adapted to their local environments can help mitigate the threat of biodiversity declines by informing conservation management strategies (Hohenlohe et al., 2021).

Global biodiversity loss has a direct negative impact on crucial ecosystem services, a prime example of which is the decline of insect pollinator species (Potts et al., 2010). According to different estimates, crop reliance on animal‐mediated pollination ranges from 78% to 94% (Ollerton et al., 2011) with bees (Hymenoptera: Anthophila) providing the majority of pollination services (Klein et al., 2007). There exist more than 20,000 species of bees (Michener, 2007) of which at least 4000 are found in North America. Recent research has shown that wild bees provide a significant proportion of pollination services both in agricultural (Kennedy et al., 2013) and urban settings (Lowenstein et al., 2015). At the community level, many plants rely on uncommon bees for pollination, highlighting the importance of rare species in plant–pollinator networks (Simpson et al., 2022).

Despite the fact that wild bee populations are particularly vulnerable to habitat loss (Kline & Joshi, 2020; Koh et al., 2016; Potts et al., 2010) as evidenced by their continued decline on the global scale (Aldercotte et al., 2022; Jacobson et al., 2018; Zattara & Aizen, 2021), bee genetic research has concentrated on a few taxa (primarily *Apis* and *Bombus* spp.), while the majority of solitary species continue to be underrepresented in conservation and genetics literature (Cameron & Sadd, 2020; Kelemen & Rehan, 2021). As sequencing becomes more accessible and is routinely used to characterize nonmodel species, newly emerging techniques and analyses make it possible to answer previously intractable conservation questions. This allows researchers to not only infer population connectivity and levels of inbreeding, but also to perform analyses that identify signatures of selection, estimate historic population sizes, and describe migration patterns (Allendorf et al., 2010; Fraser & Whiting, 2020).

In recent years, there has been an increase in the number of studies aiming to characterize local adaptation in bees. Genome‐wide screening has identified regions under selection across bee genomes (reviewed in Kelemen & Rehan, 2021); but research has predominantly focused on traits unrelated to environmental conditions (honeybees: (Cridland et al., 2018; Grozinger et al., 2007; Harpur et al., 2014; Mikheyev et al., 2015)), sweat bees: (Kocher et al., 2013), bumble bees: (Harpur et al., 2017) and carpenter bees (Rehan et al., 2016). Only a few number of studies have investigated patterns of selection with respect to geographic and climatic variation. For instance, a study on *Bombus vosnesenskii* and *B. vancouverensis*, has found an association between temperature and genes related to neural and neuromuscular function, as well as ion transport (Jackson et al., 2020). This study also found an association between precipitation and genes related to cuticle formation, homeostasis, tracheal, and respiratory system development (Jackson et al., 2020). There is also putatively adaptive genetic variation associated with latitude in the stingless bee *Melipona subnitida* (Jaffé et al., 2019) and the honeybee *Apis mellifera* (Hadley & Betts, 2012; Henriques et al., 2018). Iberian Peninsula populations of *A. mellifera* show latitudinal gradients associated with clock genes, suggesting that circadian rhythms are involved in local adaptation (Henriques et al., 2018). This species also shows distinct adaptive genetic variation along elevational gradients (Jaffé et al., 2019; Wallberg et al., 2017). Furthermore, populations of *A. mellifera* in East Africa not only exhibit panmixia but also show divergence at two haplotype blocks, one of which contains all octopamine receptor genes (Wallberg et al., 2017), which could indicate that adaptation to altitude is connected to processes associated with learning and memory.

Identification of genes under selection can provide information on molecular processes driving phenotypic divergences, as well as shed light on evolutionary trajectories responsible for local adaptations (Fraser & Whiting, 2020). As analysis of genome‐wide markers becomes a standard approach in conservation genomics, more studies aim to identify signatures of selection associated with various questions of evolutionary importance. Examples include searching for candidate genes underlying phenotypic divergence (Pimsler et al., 2017), parallel evolution (Deagle et al., 2012), adaptation to climatic conditions (Schmidt et al., 2021), and anthropogenic disturbances (Theodorou et al., 2018). Moreover, diverging loci can be used to infer patterns of genetic differentiation in recently isolated populations, in cases where conventional neutral markers do not provide enough resolution (Funk et al., 2012; Russello et al., 2012). While local adaptation patterns are largely species‐specific (Nooten & Rehan, 2020), including more species in population genomics studies can be useful for uncovering patterns in global bee declines and identifying potential conservation management strategies, by increasing our understanding of how variation in environmental conditions and historical demography affects the contemporary genetic structure of native pollinators.

Despite the recognized value of investigating evolutionary responses across a diverse array of species, population genomics studies on nonsocial bees remain scarce. Given that social bees comprise less than 10% of all bee species (Danforth et al., 2019), understanding the biology of solitary and subsocial species is crucial for evaluating the adaptive potential of bee communities in the face of changing environmental conditions. Here, we investigate whether a small carpenter bee, *Ceratina calcarata*, exhibits evidence of local adaption across its entire geographic distribution, spanning multiple climatic and elevational gradients. *Ceratina calcarata* is a common stem‐nesting bee endemic to eastern North America (Nooten et al., 2020; Rehan & Sheffield, 2011) that occurs in high abundances across various landscapes from southern Ontario in Canada to Georgia in the United States; the species is subsocial, exhibiting prolonged maternal care (Rehan & Richards, 2010). *Ceratina calcarata* is a generalist crop pollinator (Kennedy et al., 2013), among the top 20 most economically important wild bee species (Kleijn et al., 2015), and a key contributor to plant–pollinator networks (Russo et al., 2013). Given its ubiquity and the availability of genomic (Rehan et al., 2016) and transcriptomic (Rehan et al., 2014) resources, *C. calcarata* can serve as an important model species for investigating the effects of environmental variation on adaptive responses in wild bees.

Here, we employed genotyping by sequencing to collect genome‐wide data for *C. calcarata* samples across the species' entire geographic distribution. The resulting single nucleotide polymorphism (SNP) dataset was used to address several questions. First, we inferred the population structure of *C. calcarata* and compared genetic diversity estimates across different sampling locations to help characterize the ecological biogeography of the species. Additionally, we tested for evidence of adaptive divergence by identifying putative signatures of selection associated with genetic structure and environmental variation. Combined, these objectives provide insights into the population genomics of an ecologically important pollinator species, and, more broadly, demonstrate how geography and climate shape patterns of local adaptation in wild bees.

## 2 METHODS

### 2.1 Specimen collection, DNA extraction, and sequencing

**FIGURE 1:**
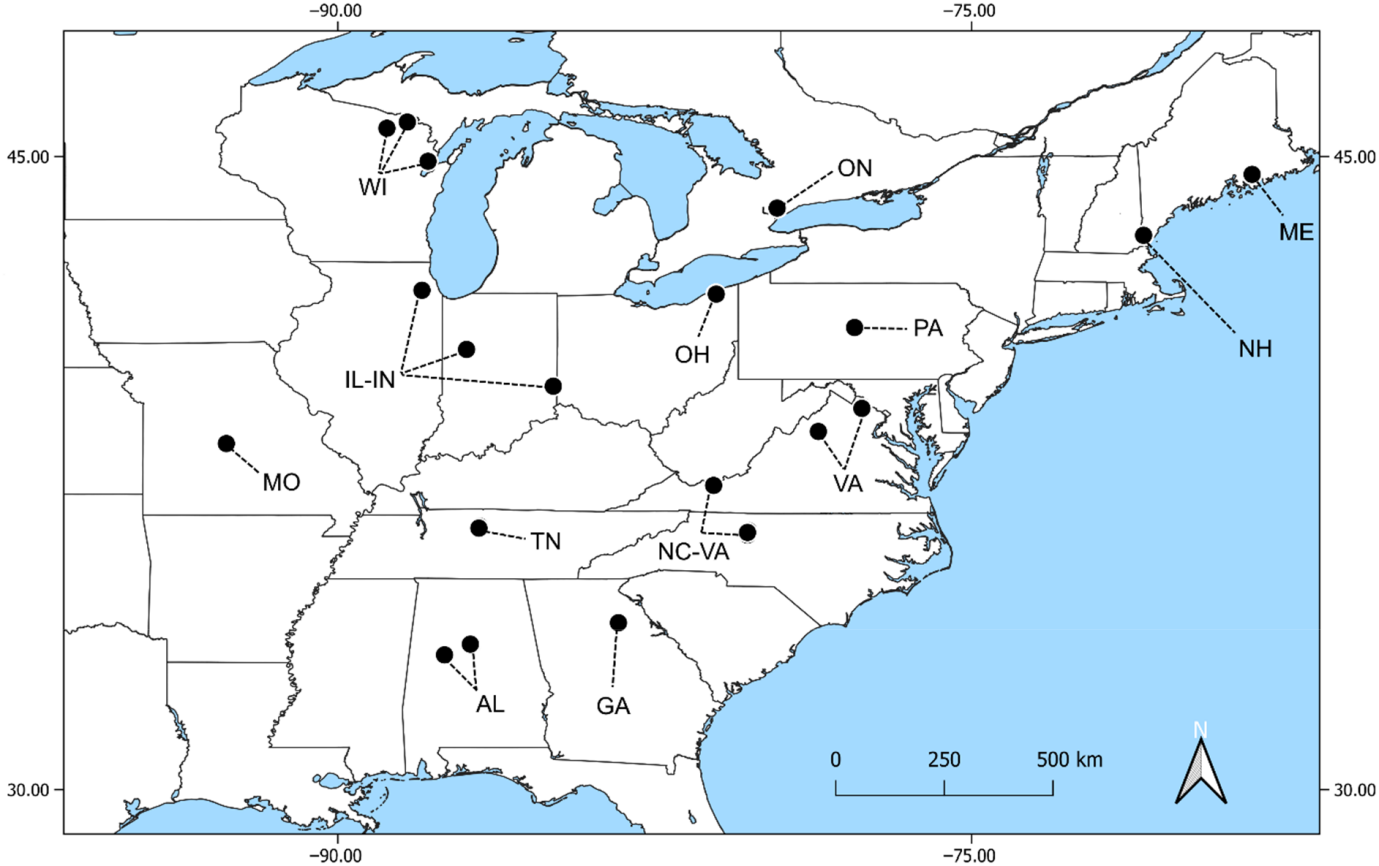
Map showing sample locations and populations of *Ceratina calcarata* examined in this study. Abbreviations of the populations, along with sample sizes are provided below. AL, Alabama (*n* = 5), GA, Georgia (*n* = 9), IL‐IN, Illinois/Indiana (*n* = 10), ME, Maine (*n* = 8), MO, Missouri (*n* = 10), NC‐VA, North Carolina/Virginia (*n* = 10), NH, New Hampshire (*n* = 8), OH, Ohio (*n* = 9), ON, Ontario (*n* = 10), PA, Pennsylvania (*n* = 10), TN, Tennessee (*n* = 5), VA, Virginia (*n* = 8), and WI, Wisconsin (*n* = 10). Map produced using QGIS.org (2020), QGIS Geographic Information System, Open Source Geospatial Foundation Project (http://qgis.org). Shape files retrieved from https://www.naturalearthdata.com/.

Adult female *C. calcarata* (*n* = 129) were collected from 33 sites from 14 regions spanning the range of the species (Figure 1; Table S1). Regions were separated by a minimum of 225 km with a maximum distance of 2787 km and a mean distance of 1079 km ± 565 SD. Bees were collected via sweep nets and pan traps in May–August 2015 to 2019 and preserved using 95% ethanol or pinning. We extracted DNA from specimens in two ways. For samples preserved in 95% ethanol, DNA was extracted from abdomens using DNeasy Blood & Tissue Kits (Qiagen). For pinned specimens, we extracted DNA from whole bodies employing a nondestructive technique (whole body incubation) using Quick‐DNA Miniprep Plus extraction kits (Zymo Research) and following a modified protocol (Freitas et al., 2021). DNA quality was assessed using agarose gel electrophoresis and a spectrophotometer (Thermo Fisher Scientific). For each individual, DNA concentration was normalized to 20 ng/μL. RAD‐seq library preparation was carried out by Floragenex, Inc, according to the original RAD protocol described by Baird et al. (2008). DNA from each individual was digested with the restriction enzyme *PstI*. Sequencing was done using two lanes of Illumina HiSeq 3000 at the Center for Genome Research and Biocomputing at Oregon State University for single pair end 100 bp reads and produced a total of 521 million reads.

### 2.2 Read mapping, variant calling, and filtering

We demultiplexed FASTQ files and removed multiplex identifying barcode sequences using *process_radtags* in STACKS *v2.3* (Catchen et al., 2011; Rochette et al., 2019). We excluded reads if they contained an ambiguous *PstI* cut site, incorrect barcode, or average Phred quality score < 10 within a sliding window (default), with 468.5 million reads (89.86%) retained following demultiplexing. Reads were filtered out because of ambiguous barcodes (9.41%), low quality scores (0.09%), or ambiguous RAD tags (0.63%). Due to a low number of reads (<7000), two individuals were removed from further analysis. Generated FASTQ files were aligned to the *Ceratina calcarata* genome (Rehan et al., 2016; BioProject: PRJNA791561) using bwa‐mem *v0.7.17* (Li & Durbin, 2009). SAM outputs were converted to BAM and sorted with SAMTOOLS *v1.6* (Li et al., 2009). SNP calling was made using *gstacks* in STACKS *v2.3* (Rochette et al., 2019) under the Marukilow model, with an alpha threshold for discovering SNPs and calling genotypes set at 0.05. We used the *populations* module in STACKS to call SNPs, present in more than 80% of individuals across all populations (‐*R 80*), minimum minor allele frequency of 0.05 (*‐‐min‐maf* 0.05), maximum observed heterozygosity of 0.5 (‐*‐max‐obs‐het 0.5*), and retaining one SNP per locus (‐*‐write‐single‐snp*) to decrease the effects of linkage disequilibrium. Following the initial *populations* run, we identified and removed low coverage individuals (depth < 6×) and/or individuals with high missing data percentage (>50%), calculating both statistics using vcftools *v0.1.16* (Danecek et al., 2011). After removing 15 samples due to low coverage and/or high missing data percentage, we reran *populations* on the remaining samples, using the same parameters as above. We used vcftools *v0.1.16* to filter the resulting VCF file, keeping SNPs with minimum depth of 10x and maximum depth of 100× (‐‐*min‐meanDP 10* and ‐‐*max‐meanDP 100*) and missing data below 10% (‐*‐max‐missing 0.9*). To ensure that no potential sibling pairs were present in the dataset, we calculated relatedness using the kinship coefficient method (Manichaikul et al., 2010), as implemented in vcftools *v0.1.16* (‐*‐relatedness2*). The resulting dataset was used to identify outlier SNPs.

### 2.3 Detection of outlier SNPs

Genome scans are used in population genomics studies to identify potentially divergent loci; however, this approach is characterized by a high level of false positives (Narum & Hess, 2011). To mitigate this issue, a common strategy is to use several outlier detection methods (Alves‐Pereira et al., 2020). Here, we combined four different approaches. First, we employed the *F*_ST_‐based method implemented in Bayescan v2.0 (Foll & Gaggiotti, 2008), running the program for 100,000 iterations with a 50,000 burn‐in period and prior‐odds set to 100. SNPs with a *q* ˂0.05 were considered outliers.

Second, we detected outliers using the principal component analysis (PCA) method, as implemented in *pcadapt v4.3.3* (Luu et al., 2017). Using Cuttel's rule, we inferred the optimal number of principal components (PC), and then employed the *ld. clumping* procedure to thin the SNP dataset, with the window radius of 200 and the squared correlation threshold of 0.2. We corrected the resulting *p*‐values for multiple comparisons using the method of Benjamini and Yekutieli (2001), using the R package *q*‐value, assuming all SNPs with a *q* <0.05 to be outliers.

Third, to identify signatures of local adaptation potentially associated with environmental variables, we used the gene–environment association (GEA) approach for outlier loci detection. We selected the following geographical/environmental variables for this analysis: latitude, longitude, elevation, mean annual temperature, and mean annual precipitation between the years 1970–2000 (https://www.worldclim.org/data/worldclim21.html). For each sample in the dataset, we extracted environmental variables based on sample coordinates, using the R package *raster v3.4–5*. To control for multicollinearity, we conducted a collinearity test between potential environmental predictors using the *cor* command within R package *stats v4.04* and removed one variable from each pair, where collinearity estimate (*r*) was higher than 0.8. Based on this test, temperature was highly correlated with latitude, and hence, only one of these variables (temperature) was kept as a predictor for GEA tests. First, we used latent factor mixed modelling (LFMM), as implemented in the R package *LEA* (Frichot & François, 2015). This method uses individual level data to find associations between allele frequencies and environmental variables of interest, and accounts for background population structure using latent factors. To select the optimal number of latent factors for this analysis, we estimated admixture coefficients using Sparse nonnegative matrix factorization (SNMF), selecting a number of clusters (K) with the lowest cross entropy. We then imputed missing data using the *impute* function in the LEA package, using the selected value of *K*. LFMM was run with 10 repetitions, 100,000 iterations and a burn‐in step of 50,000. Finally, we combined the resulting z‐scores across all runs, and recalculated *p*‐values, using the genomic inflation factor (*λ*). Following *λ* recalibration and a subsequent correction for multiple comparisons, SNPs with a *q*‐value below 0.05 were considered outliers.

Finally, we used another GEA approach implemented in BayPass (Gautier, 2015), which identifies loci that are associated with population‐specific covariates. To account for the shared ancestry and population structure within the dataset, we used the core model within BayPass to generate a covariance matrix of allele frequencies. The resulting matrix was then used as an input file to run the auxiliary covariate model, which tests for associations between SNPs and population covariates. We ran both models for 20 pilot runs, 10,000 iterations, and a burn‐in period of 50,000. SNPs with a Bayes factor above 20 dB were considered outliers.

### 2.4 Annotation of outlier SNPs

We extracted shared SNPs among the above four methods, using the R packages *dplyr* and *tidyft* within *tidyverse v1.3.1* (Wickham et al., 2019) and *gplots v3.1.1* (Warnes et al., 2020): SNPs that were identified as outliers by two or more methods were considered robust. We used bedtools v2.26.0 (Quinlan & Hall, 2010) and BEDOPS v2.4.38 (Neph et al., 2012) *bedmap ‐‐echo‐map‐id‐uniq* command to extract gene IDs associated with genomic positions of the robust outlier SNPs, using the latest annotation of the *C. calcarata* genome (http://www.rehanlab.com/genomes.html PO1409_Ceratina_calcarata.annotation.gff.gz). We investigated the putative role of these genes, using the R package *TopGO* (Alexa & Rahnenfuhrer, 2022), by testing the associated gene ontology (GO) terms (terms generated by BLAST2GO (Gotz et al., 2008)), for enrichment using the *elim* algorithm and Fisher's exact test to test for significance. Only biological process (BP) terms were used in this analysis and associations with a *p*‐value below 0.05 were considered significant.

### 2.5 Population genetics analyses

To create a putatively neutral SNP dataset for downstream population genetics analyses, we removed all SNPs that were identified as outliers by at least one of the above methods. Furthermore, we removed SNPs that significantly (*p* < 0.01) deviated from Hardy–Weinberg equilibrium (HWE) in at least 50% of the populations. The resulting SNP dataset was used for all population genetics analyses below, unless otherwise specified.

First, we calculated observed (*H*_O_) and expected (*H*_S_) heterozygosities, observed and effective numbers of alleles, and inbreeding coefficients (*G*_IS_) for each sampling location in the dataset using GenoDive v3.04 (Meirmans, 2020). We estimated pairwise differentiation between sampling locations by calculating θ (Weir & Cockerham, 1984), as implemented in GenoDive v3.04, using 10,000 permutations to test for significance.

To evaluate the population structure of *C. calcarata*, we used the Bayesian clustering approach, as implemented in STRUCTURE v3.4 (Pritchard et al., 2000). Run length was set to 100,000 Markov chain Monte Carlo (MCMC) replicates after a burn‐in period of 100,000 using correlated allele frequencies under an admixture model using the LOCPRIOR option. The number of clusters (*K*) was varied from 1 to 14, with 10 iterations of each. To infer the optimal *K* value, we used the Δ*K* method (Evanno et al., 2005), summarized using STRUCTURE HARVESTER (Earl & vonHoldt, 2012). We also plotted the log probability of the data to assess where ln Pr(X|K) plateaued (Pritchard et al., 2000). Lastly, we visualized the output, using CLUMPAK (Kopelman et al., 2015). To further examine the population structure not revealed by neutral loci, we conducted a separate STRUCTURE analysis using only robust outlier SNPs and the same parameters as above. While usage of SNPs that are not in HWE violates one of the key assumptions of STRUCTURE, some studies suggest that population structure identified by outlier loci can help identify adaptive divergence patterns not found by analysis of neutral SNPs alone, but which might be important for informing conservation management decisions (Funk et al., 2012; Milano et al., 2014; Schmidt et al., 2021).

Additionally, we examined population structure using TESS3 (Caye et al., 2016), which incorporates spatial information into the calculation of ancestry coefficients. We ran the program as implemented in the associated R package, using the default parameters and varying the number of clusters from 1 to 10. To choose the optimal number of clusters, we examined the cross‐validation scores, to identify where the plot exhibited a plateau. To further evaluate population differentiation, we conducted a PCA using SNPRelate v1.14.0 (Zheng et al., 2012) on both neutral (*n* = 6517) and robust outlier (*n* = 250) SNPs and visualized the results using R packages *plotly v4.9.0* (Sievert, 2020) and *ggplot2 v3.3.5* (Wickham et al., 2019).

## 3 RESULTS

### 3.1 Population genetics analyses

After full filtering, 112 individuals and 8302 SNPs were retained in the dataset, with an average depth of 55.16× and mean missing data percentage of 6.03%. Following the removal of unique outliers, no SNPs were out of HWE: therefore, 6517 putatively neutral SNPs were retained for population genetics analyses. Across all sampling locations, observed heterozygosity was lower than expected heterozygosity (Table 1) and ranged from 0.074 (VA) to 0.164 (ON). The overall inbreeding coefficient across populations was significantly different than 0 at 0.336 (95% CI: 0.329–0.342), ranging from 0.037 (ON) to 0.701 (VA) (Table 1). Based on calculations of pairwise genetic differentiation between sampling locations, WI population was most highly differentiated from all other sampling locations (Table 2).

**TABLE 1:** Population diversity metrics calculated for all sampling locations (populations) based on 6517 putatively neutral SNPs, as estimated using GenoDive v3.04 (Meirmans, 2020).

Population	*N*	*N*_A_	*N*_E_	*H*_O_	*H*_S_	*G*_IS_
AL	5	1.538	1.275	0.123	0.205	0.400 (*p* < 0.01)
GA	9	1.6	1.243	0.161	0.169	0.047 (*p* < 0.01)
IL‐IN	10	1.883	1.333	0.153	0.24	0.362 (*p* < 0.01)
ME	8	1.387	1.195	0.075	0.141	0.465 (*p* < 0.01)
MO	10	1.83	1.276	0.149	0.202	0.263 (*p* < 0.01)
NC‐VA	10	1.766	1.273	0.152	0.197	0.230 (*p* < 0.01)
NH	8	1.527	1.229	0.115	0.161	0.285 (*p* < 0.01)
OH	9	1.607	1.245	0.159	0.17	0.064 (*p* < 0.01)
ON	10	1.63	1.246	0.164	0.17	0.037 (*p* < 0.01)
PA	10	1.595	1.227	0.125	0.161	0.225 (*p* < 0.01)
TN	5	1.686	1.403	0.129	0.283	0.544 (*p* < 0.01)
VA	8	1.692	1.352	0.074	0.249	0.701 (*p* < 0.01)
WI	10	1.647	1.31	0.11	0.207	0.470 (*p* < 0.01)

Abbreviations: *G*_IS_, inbreeding coefficient; *H*_O_, observed heterozygosity; *H*_S_, expected heterozygosity; *N*, sample size; *N*_A_, number of alleles; *N*_E_, number of effective alleles.

**TABLE 2:** Estimates of pairwise *θ* (Weir & Cockerham, 1984) between all pairs of populations (above the diagonal), with their associated *p*‐values (below the diagonal), as estimated using GenoDive v3.04 (Meirmans, 2020).

	AL	GA	IL‐IN	ME	MO	NC‐VA	NH	OH	ON	PA	TN	VA	WI
AL	–	0.025	−0.014	0.035	−0.005	0.001	0.02	0.032	0.031	0.025	0.058	−0.012	0.373
GA	0.002	–	0.03	0.039	0.01	0.002	0.014	0.02	0.016	0.012	0.178	0.121	0.458
IL‐IN	0.505	0.128	–	0.031	−0.013	0.001	0.02	0.026	0.029	0.025	0.016	0.003	0.284
ME	0.01	0	0.218	–	0.013	0.024	0.018	0.04	0.026	0.028	0.175	0.12	0.473
MO	0.384	0	0.218	0.008	–	−0.008	0.001	0.01	0.006	0.005	0.091	0.056	0.378
NC‐VA	0.145	0.16	0.211	0.001	0.475	–	0.007	0.012	0.012	0.006	0.116	0.074	0.399
NH	0.039	0	0.327	0	0.432	0.019	–	0.013	0.006	0.006	0.171	0.115	0.461
OH	0.001	0	0.204	0	0	0	0	–	0.012	0.007	0.177	0.123	0.459
ON	0	0	0	0	0	0	0.001	0	–	0.005	0.184	0.127	0.462
PA	0.023	0	0.225	0	0	0.001	0.009	0.001	0.001	–	0.179	0.121	0.465
TN	0.182	0.008	0.311	0.034	0.043	0.028	0.028	0.008	0.002	0.023	–	−0.049	0.106
VA	0.495	0	0.315	0.045	0.081	0.06	0.004	0	0	0.002	0.666	–	0.197
WI	0.003	0.001	0.004	0	0	0	0	0	0	0	0.086	0.014	–

**FIGURE 2:**
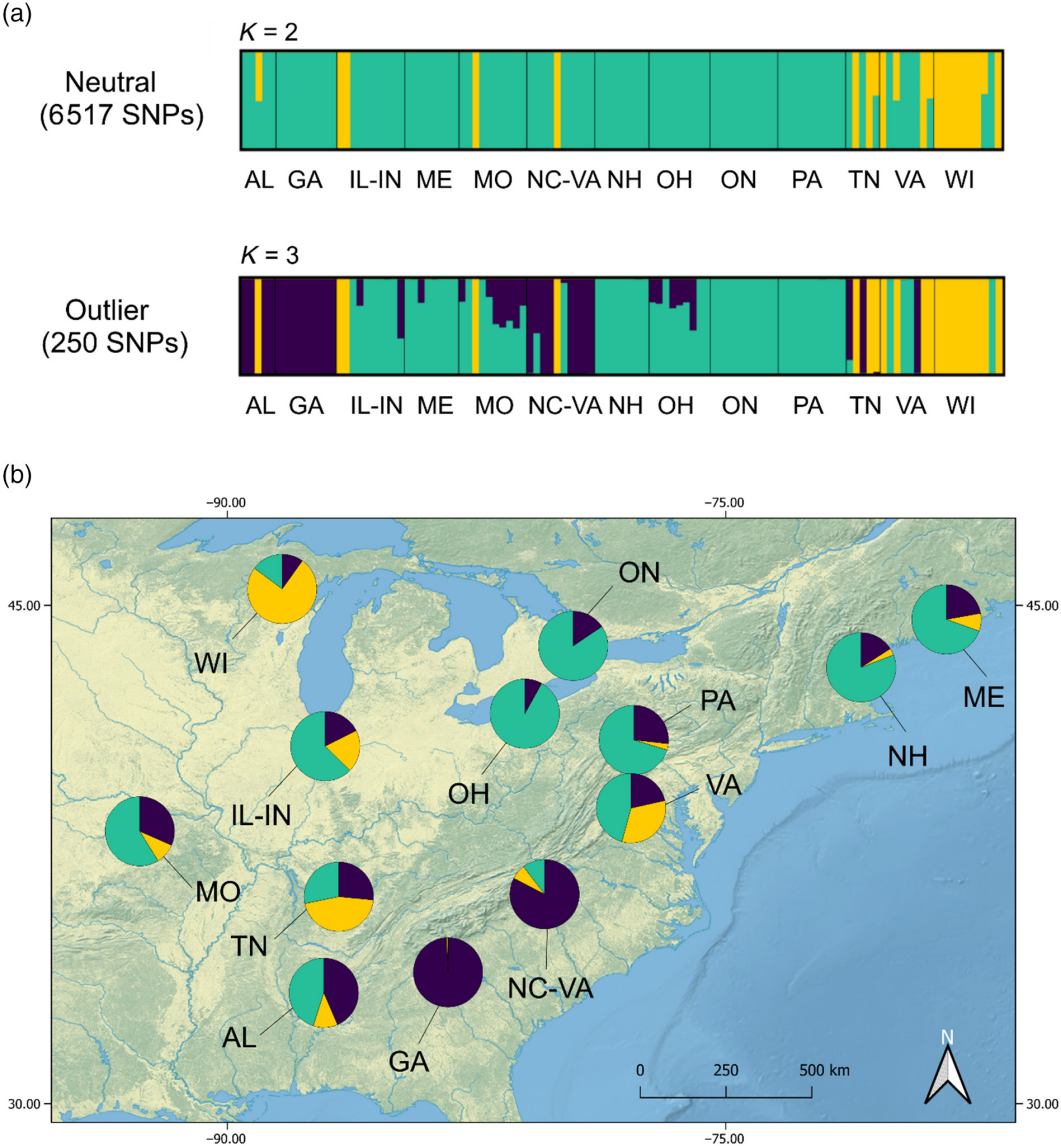
(a) Results of the Bayesian clustering method, as implemented in STRUCTURE v3.4 (Pritchard et al., 2000). Output results represent the optimal *K* value (*K* = 2 or *K* = 3), as determined by the Δ*K* method (Evanno et al., 2005), using STRUCTURE HARVESTER (Earl & vonHoldt, 2012). Visualized using CLUMPAK (Kopelman et al., 2015). (b) Results of the *K* = 3 clustering generated by TESS3, based on 6517 putatively neutral SNPs. Map produced using QGIS.org (2020), QGIS Geographic Information System, Open Source Geospatial Foundation Project (http://qgis.org). Shape files retrieved from https://www.naturalearthdata.com/.

**FIGURE 3:**
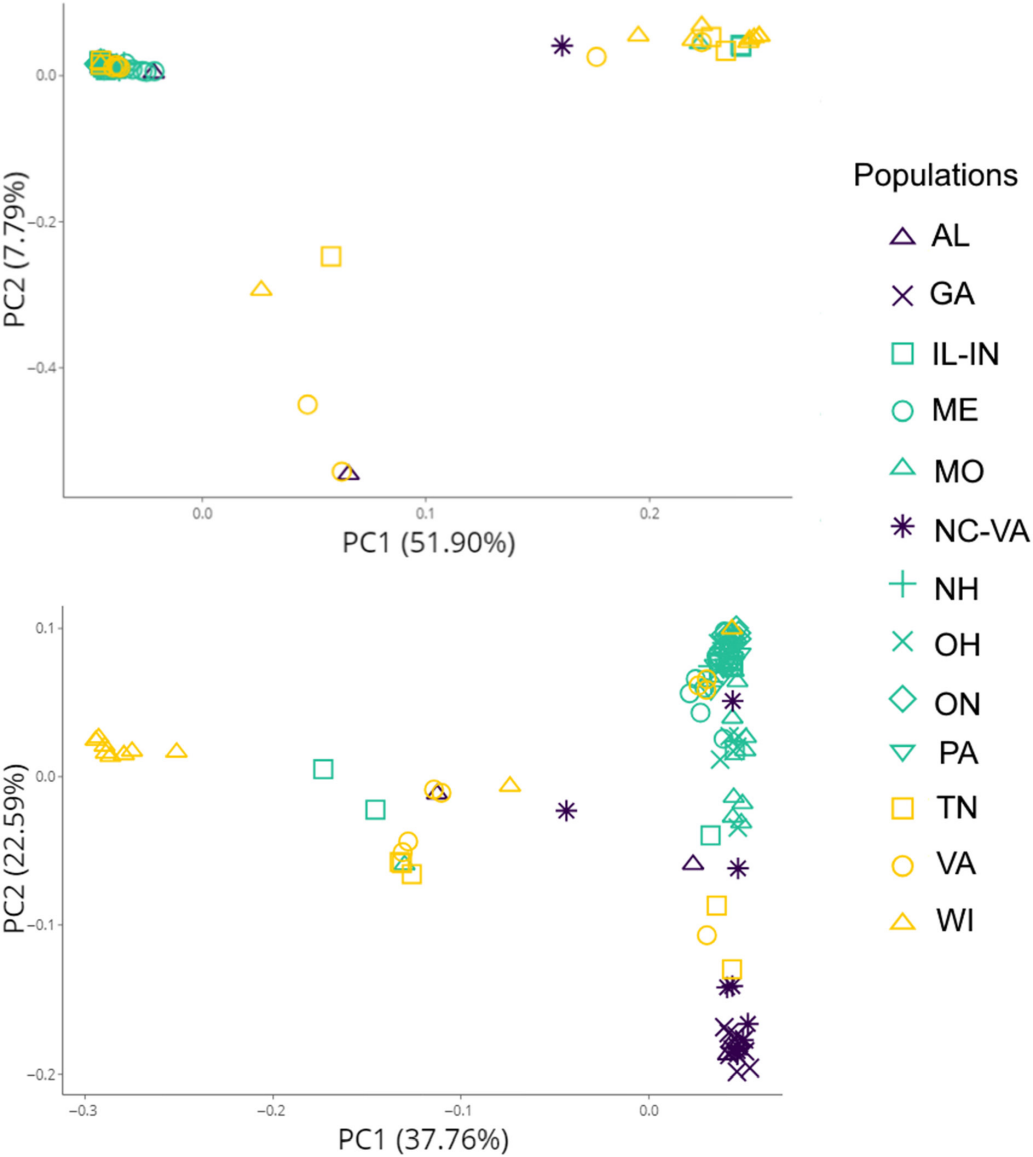
Principal component analysis (PCA) for 112 *Ceratina calcarata* samples across 13 populations, produced using 6517 putatively neutral SNPs (top panel) or 250 robust outlier SNPs (bottom panel). PCA analysis was conducted using SNPRelate v1.14.0 (Zheng et al., 2012).

The STRUCTURE analysis based on the putatively neutral SNPs revealed evidence for two clusters that best explained the genetic variation within the dataset (Figures 2a, S3). The first cluster was mostly formed by WI samples, and included some individuals from TN, VA, IL‐IN, MO, and NC‐VA, while the second cluster included all the remaining populations. Further, STRUCTURE analysis based on the robust outlier SNPs showed highest support for *K* = 3 (Figure S3), separating the southern populations (AL, GA, NC‐VA, and MO) from the original cluster. While TESS3 analysis was inconclusive between *K* = 2 and *K* = 3, the clustering results revealed by TESS3 largely agreed with those found by STRUCTURE (Figure 2b). The PCA on the neutral dataset supported the results of the clustering programs, with the first two principal components separating WI from other populations in the dataset; PCA on the robust outlier SNPs revealed further divergence of the southern populations (Figure 3).

### 3.2 Outlier detection and annotation of outlier SNPs

BayeScan detected 90 SNPs, while *pcadapt*, LFMM, and BayPass detected 884, 887, and 251 outlier SNPs, respectively (Table S2, Figure S1). All 90 outliers identified by BayeScan were under diversifying selection (alpha >0; Figure S2). LFMM identified 429 outliers associated with longitude, 402 with temperature, 183 with precipitation, and 150 with elevation: of these, 250 outliers were associated with two or more variables. Out of the 251 BayPass outliers, 50 were associated with longitude, 144 with temperature, 37 with precipitation, and 53 with elevation: 33 outliers were associated with two or more environmental variables. Combined, we detected 1784 unique outliers, of which 23 SNP was detected in common by all four methods, 32 SNPs were detected in at least three out of four methods, and 250 were detected by two or more methods, and thus considered robust (Table S2, Figure S1). Of the 250 robust outliers, 70 were associated with longitude by at least one of the GEA analyses, 171 were associated with temperature, 91 with precipitation, and 39 with elevation (Tables S2, S3). When comparing robust outliers by covariables, LFMM and BayPass shared 39 outliers associated with longitude, 134 outliers associated with temperature, 31 outliers associated with precipitation, and 17 outliers associated with elevation.

Annotation against the *C. calcarata* genome revealed that 250 robust outliers were mapped against 126 unique (143 total) gene identifiers, of which 97 (Table S3) were from genes of known function. Of the 134 temperature‐associated SNPs detected by both LFMM and BayPass, annotations were available for 80 SNPs, including eye‐specific diacylglycerol kinase (*rdgA*), a gene crucial for photoreceptor maintenance (Masai et al., 1997); multidrug resistance protein homolog 49 (*Mdr49*), which contributes to insecticide resistance; synaptic vesicle membrane protein VAT‐1 homolog‐like (*Vat1l*)—a gene expressed in brains of a variety of taxa from zebrafish to humans (Loeb‐Hennard et al., 2004). Several outlier SNPs exhibited divergence patterns that could be linked to geographic and climatic factors. For example, one SNP (Scaffold 3_12741967; hereafter, the number after the underscore refers to the SNP position within the scaffold) that was detected as an outlier associated with longitude, temperature, and elevation (Table S3) annotated to ATP‐dependent 6‐phosphofructokinase (*Pfk*), which is involved in glycometabolism (Leite et al., 1988); at this SNP, New Hampshire and Maine had the same genotype in contrast to all other populations. Furthermore, three outlier SNPs (Scaffold 1_18492518; Scaffold 1_18495934; Scaffold 1_18401015) found in association with longitude, temperature, and precipitation were located on Scaffold 1 and annotated to steroid receptor seven‐up (*svp*) that has a role in eye development (Tsachaki & Sprecher, 2012), and *king‐tubby*—a gene known for its function in retinal degeneration (Carbone et al., 2016). At two of these three SNPs, southern populations (Alabama, Georgia, North Carolina‐Virginia, and Missouri) were differentiated from the rest of the populations. Lastly, across the 126 candidate genes, TopGO analysis identified 43 significantly enriched GO terms (Table S4), including those related to cellular response to UV, cellular response to light stimulus, and disruption by virus of host envelope lipopolysaccharide during virus entry.

## 4 DISCUSSION

The loss of native pollinator abundance and diversity has wide‐ranging impacts on natural ecosystems (Potts et al., 2010), agriculture, and economy (Garibaldi et al., 2013; Potts et al., 2010). However, little is known about the genetics of wild bees and molecular mechanisms that enable their ability to adapt to environmental conditions. Here, we investigate the population genomics of an important native pollinator across its entire geographic distribution. The results of our data analysis demonstrate that *C. calcarata* population is separated into multiple genetic clusters, reflecting the geographical features of the species' range across eastern North America. Identification of putative signatures of selection revealed outlier loci involved in molecular processes potentially contributing to local adaptation, including thermoregulation, light sensitivity, stress response, and immunity.

### 4.1 Patterns of genetic differentiation and inbreeding

The results of several population structure analyses conducted on a genome‐wide set of SNPs were consistent with the presence of two to three genetic clusters within *C. calcarata*. The majority of the samples (Maine, New Hampshire, Ontario, Ohio, and Pennsylvania) belonged to the Northeastern genetic cluster; the Wisconsin samples formed a Western cluster, while Georgia and North Carolina‐Southern Virginia samples comprised the Southern cluster (Figures 2, 3). Alabama, Illinois‐Indiana, Tennessee, Missouri, and Virginia samples appeared to be highly admixed, and hence are not included as part of the three clusters.

We found strong evidence for differentiation of the Wisconsin population, as it was highly diverged from the rest of the samples (Table 2), forming a separate genetic cluster based on the analysis of both neutral and putatively adaptive SNPs (Figures 2, 3). The longitudinal differentiation was also reflected in the admixture shown by samples collected in the midwestern part of the study area (i.e., Illinois‐Indiana, Missouri, Tennessee, and Virginia). This clustering suggests a West to East divergence of *C. calcarata*: a pattern also observed in other species that could be indicative of phylogeographical breaks (Soltis et al., 2006). For instance, molecular studies on plants propose that climatic fluctuations during the Pleistocene reduced the available range for many species, forcing them to occupy glacial refugia and subsequently diverge after the last glacial maximum ~20,000 years ago (Hewitt, 1996; Ony et al., 2021). Previous research has indicated a role for the Appalachian Mountains in postglaciation expansions: historically, the mountain range could have acted as a barrier to dispersal (Soltis et al., 2006). This discontinuity has been investigated for many animal and plant species (Austin, 2002; Church et al., 2003; Vickruck & Richards, 2017; Zamudio & Savage, 2003). For instance, in a generalist pollinator *Xylocopa virginica*, Iowa and Arkansas populations are highly differentiated from the core genetic cluster located in the Eastern part of the continent (Vickruck & Richards, 2017). Several studies have investigated postglaciation divergence in North America and effects of climate on range expansions focusing on plants (Hoban et al., 2010; McLachlan et al., 2005). For example, in eastern redbud *Cercis canadensis* population, differentiation occurs along the Appalachian Mountains (Ony et al., 2021), a genetic split comparable to that exhibited by *X. virginica*. It is probable that other pollinator species have followed similar recolonization routes. A phylogeographical analysis of the five eastern North American *Ceratina* species showed that they have experienced a population expansion approximately 20,000 years ago coinciding with glacial retreat (Shell & Rehan, 2016). Following angiosperms that were able to occupy new suitable habitats as the ice gradually retreated (Hewitt, 1996), insect populations started a similar recolonization process (Shell & Rehan, 2016). The postglaciation refugia hypothesis has been examined on other continents: for example, a pollen‐specialist bee *Colletes gigas* endemic to China was shown to exhibit fluctuations in effective population size during the last glacial maximum (Su et al., 2022).

Another signature of a recolonization process following glaciation is reduced genetic diversity along the dispersal routes and higher differentiation of the peripheral clusters. In accord with this prediction, *C. calcarata* exhibits high levels of inbreeding across its entire geographic range (Table 1). According to the central‐marginal hypothesis, populations located toward the periphery of the species' range exhibit lower levels of genetic diversity (Eckert et al., 2008). For example, increased differentiation and decreased genetic diversity in marginal populations have been observed in another *Ceratina* species, *C. australensis*, where levels of inbreeding were elevated in the recently established southern populations, but not in the more genetically diverse and older Queensland population (Harpur & Rehan, 2021). Similarly, we observe that the level of inbreeding in the Western (Wisconsin) cluster is higher (*G*_IS_ = 0.47) than the average level of inbreeding in the Northeastern (mean *G*_IS_ = 0.30 ± 0.18) or the Southern genetic cluster (mean *G*_IS_ = 0.14 ± 0.09). However, given the variation in sample size between different locations in this study, this comparison should be interpreted with caution. Additionally, we did not find any evidence for substructure within the Northeastern cluster; despite this region spanning a large geographic distance, there was little genetic differentiation between sampling locations. This pattern could be attributed either to high gene flow between these locations or indicate that the founding population was characterized by low levels of genetic diversity. Bee foraging distance is correlated with body size (Gathmann & Tscharntke, 2002): given the small body size of *C. calcarata*, it is unlikely that the absence of population structure in this region is due to recent gene flow and more probably attributed to the founder effect after glaciation. Finally, results of the genetic clustering analyses using robust outlier SNPs reveal additional patterns of differentiation providing support for the separation of the Southern populations. This divergence might indicate that *C. calcarata* populations are undergoing local adaptation, reflective of current or historic climatic processes.

### 4.2 Signatures of selection

Studies of local adaptation help to disentangle interactions between various evolutionary forces and environmental heterogeneity, contributing to our understanding of speciation and natural selection at a broader scale. These findings can be used by conservation biologists to identify current and future threats to economically important or critically endangered populations. Here, we used a genome scan approach to identify signatures of differential selection in a wild pollinator, focusing on its entire geographic distribution. We identified putatively divergent SNPs associated with population structure, as well as geographic and climatic variation. The results revealed several genes with potential relevance to bee physiology and response to external stressors, providing clues into gene–environment interactions in wild bees more generally. While this study represents a starting point in understanding the patterns of divergence in *C. calcarata*, we recognize that reduced representation sequencing methods (such as RAD‐seq) capture only a fraction of the genome and likely underestimate the true number of divergent loci (Lowry et al., 2017). Thus, a more thorough understanding of the local adaptation in this species would require a follow‐up whole‐genome analysis.

In our study, we identified a number of putatively selected genes, potentially linked to thermal regulation (Tables 3, S3). For instance, one annotated gene (*pfk*) was related to glycolysis regulation, and more specifically to the phosphofructokinase/fructose‐l,6‐bisphosphatase‐mediated substrate cycle (Leite et al., 1988). In bumblebees, this substrate cycle is critical for flight muscle activity, and is dependent on environmental temperature (Leite et al., 1988). Interestingly, at the SNP located on *pfk* gene, New Hampshire and Maine samples show similar genotypes. Within our dataset, Maine and New Hampshire are characterized by colder temperatures: this is in line with the prediction that selection on genes involved in thermoregulation will result in divergent genotypes in populations found in different climatic conditions. A study on bumblebees (Liu et al., 2020) found that *Pfk1* was among the 19 genes that were upregulated in populations inhabiting high altitude conditions, further highlighting the role of this gene in cold tolerance. Another outlier SNP in our study annotated to facilitated trehalose transporter (*Tret1‐like*), which is involved in the uptake of trehalose—sugar circulating in insect hemolymph—which plays a role in energy metabolism and stress recovery (Shukla et al., 2015). Additionally, one of the robust outlier SNPs annotated to inositol monophosphatase 3 (*CG15743*): in eukaryotic cells, inositol monophosphatase is an enzyme involved in myo‐inositol synthesis (Michell, 2008). Myo‐inositol has been shown to increase in overwintering *D. melanogaster*, suggesting the enzyme's role in cold resistance (Vesala et al., 2012). Previous studies found that genes involved in sugar metabolism and energy biosynthesis are differentially expressed in bees subject to extreme temperature conditions (Liu et al., 2020; Xu et al., 2017); hence, it is expected that these genes will show signatures of divergent selection in populations distributed across a climatic gradient. Also, of interest are outlier SNPs annotated to ATPase genes—katanin p60 ATPase‐containing subunit A‐like 1 (*katnal1*) and calcium‐transporting ATPase type 2C member 1 (*Atp2c1*), which have been associated with climate adaptations (Eydivandi et al., 2021; Yu et al., 2021). While most insects are ectotherms, many, including bees, exhibit thermoregulatory behavior, making them facultatively endothermic (Heinrich, 1975; Stone & Willmer, 1989). This ability makes it possible for bees to function under different climatic conditions, inhabiting large geographic regions (Heinrich, 1975). Thermoregulation in bees is critical for flight, efficient foraging, and young rearing (Heinrich, 1975; Kovac & Schmaranzer, 1996; Somanathan et al., 2019); therefore, selection is predicted to act on genes that are tightly linked to functions associated with thermal responses. Two temperature‐associated outlier SNPs annotated to genes with a role in neural development (*Vat1l*, *OTOF*); furthermore, one of the significantly enriched GO terms (GO:0007616) was associated with long‐term memory. These findings are consistent with previous research that revealed genes involved in neural and neuromuscular processes to be under selection in other bee species, across temperature (Jackson et al., 2020), and elevation gradients (Wallberg et al., 2017).

**TABLE 3:** A subset of robust outlier SNPs identified in this study and their associated annotations. For the complete list of SNPs (*n* = 250) and annotations, see Table S3.

	Scaffold	SNP	Gene ID	Gene description
Thermoregulation and energy metabolism	Scaffold_3	12,741,967	Ccalc.v3.03962	Similar to Pfk: ATP‐dependent 6‐phosphofructokinase (*Drosophila melanogaster*)
	Scaffold_7	3,528,284	Ccalc.v3.06845	Similar to Tret1: Facilitated trehalose transporter Tret1 (*Apis mellifera ligustica*)
	Scaffold_14	53,709	Ccalc.v3.13777	Similar to Atp2c1: Calcium‐transporting ATPase type 2C member 1 (*Rattus norvegicus*)
	Scaffold_3	12,172,435	Ccalc.v3.03863	Similar to katnal1: Katanin p60 ATPase‐containing subunit A‐like 1 (*Danio rerio*)
	Scaffold_7	1,457,239	Ccalc.v3.06639	Similar to CG15743: Putative inositol monophosphatase 3 (*Drosophila melanogaster*)
Photoperiod	Scaffold_1	18,241,238	Ccalc.v3.02814	Similar to rdgA: Eye‐specific diacylglycerol kinase (*Drosophila melanogaster*)
	Scaffold_1	18,492,518	Ccalc.v3.02835	Similar to svp: Steroid receptor seven‐up, isoforms B/C (*Drosophila melanogaster*)
	Scaffold_1	18,401,015	Ccalc.v3.02826	Similar to king‐tubby: Protein king tubby (*Drosophila persimilis*)
	Scaffold_3	816,030	Ccalc.v3.02948	Similar to UVOP: Opsin, ultraviolet‐sensitive (*Apis mellifera*)
Response to stress	Scaffold_8	8,436,752	Ccalc.v3.10243	Similar to Mdr49: Multidrug resistance protein homolog 49 (*Drosophila melanogaster*)
Neural development	Scaffold_5	173,692	Ccalc.v3.08566	Similar to Vat1l: Synaptic vesicle membrane protein VAT‐1 homolog‐like (*Mus musculus*)
	Scaffold_1	18,444,442	Ccalc.v3.02831	Similar to OTOF: Otoferlin (*Homo sapiens*)

In our study, we also identified several genes (*svp*, *king‐tubby*, and *rdgA*), which play a role in the development of photoreceptor cells (Tables 3, S3). In a study on *D. melanogaster* senescence, *king‐tubby* was among the top candidate genes that promote visual decline (Carbone et al., 2016). Selection on genes that regulate eye development could be driven by variation in daylight amount among various locations in our study. For instance, our results show Alabama, Georgia, North Carolina, and Missouri populations show similar genotypes at genes associated with eye development—this could be explained by a difference in photoperiod length experienced by populations at Southern latitudes. Together with temperature, photoperiod is known to impact many critical bee life‐history traits, such as the timing of brood onset (Bennett et al., 2018; Nürnberger et al., 2018) and diapause regulation (Pitts‐Singer, 2020).

In addition to genes linked to thermal regulation and light sensitivity, we also found outlier SNPs putatively associated with adaptation to various biotic and abiotic threats. Of the 43 significantly enriched GO terms, three (GO:0034644: cellular response to UV; GO:0071482: cellular response to light stimulus; GO:0009416: response to light stimulus) were linked to biological processes associated with light responses. Exposure to ultraviolet (UV) light constitutes a type of oxidative stress; in honeybees, nurse bees subject to UV radiation show an elevated expression of heat shock proteins in abdomens and heads, which is a marker typically indicative of thermal stress (Kim et al., 2019). Likewise, in a solitary bee *Osmia bicornis*, exposure to UV light was found to cause body deformities and increased mortality (Wasielewski et al., 2015). Another outlier SNP annotated to multidrug resistance protein homolog 49 (*Mdr49*), which could be a signature of selection related to increased pesticide usage. Research has shown that interaction with certain compounds commonly found in bee habitats (e.g., fumagillin), can inhibit multidrug resistance transporters, increasing honeybee sensitivity to ivermectin and acetamiprid (Levy & Marshall, 2013). Finally, one of the significantly enriched GO terms (GO:0098995: disruption by virus of host envelope lipopolysaccharide during virus entry) was related to immune function. Both managed and wild bees are highly susceptible to pathogenic infections (Mallinger et al., 2017), making immune genes a highly probable target of selection pressure. This finding is congruent with previous studies that detected immune‐related genes showing signatures of selection in other bee species, such as *A. mellifera iberiensis* (Chávez‐Galarza et al., 2013) and *B. terricola* (Kent et al., 2018).

## 5 CONCLUSION

Combined, our data show how geographic and climatic features shape genetic structure and diversity of natural populations and provide important insights into mechanisms by which native pollinator insects adapt to environmental conditions, using *C. calcarata* as an example. We demonstrate that despite its widespread occurrence, this wild pollinator exhibits decreased heterozygosity and elevated levels of inbreeding. Furthermore, our results provide evidence that patterns of neutral divergence in *C. calcarata* are associated with longitude, forming two main genetic clusters; at the same time, evaluation of adaptive divergence reveals a third genetic cluster in the Southern part of the continent. Analysis of signatures of selection shows that *C. calcarata* is undergoing geography‐ and climate‐associated adaptation, with molecular processes linked to thermoregulation, abiotic and biotic stress, and light sensitivity being under putatively divergent selection. Finally, our work highlights the utility of a small carpenter bee as a potential model species for evaluating the interactions between genetics and environment. As anthropogenic disturbances and global warming continue to modify critical pollinator habitats, wild bees are going to be disproportionally affected. Continuous monitoring of the genetic diversity, structure and adaptive processes of vulnerable populations is going to be vital in determining the resilience potential of bee communities as a whole.

## Supporting information


Figure S1.

Table S1.

## Data Availability

Sequence data are publicly available in NCBI, BioProject: PRJNA791561. Reference genome, annotation, and gene ontology files used in this study are available at http://www.rehanlab.com/genomes.html.
