# Supplementary material for: The impact of geography and climate on the population structure and local adaptation in a wild bee
Source: Evol Appl. 2023 May 8;16(6):1154–68. doi: 10.1111/eva.13558 (PMC10286232; doi:10.1111/eva.13558)
Supplement: Supplementary file 1 — Figure S1. [file EVA-16-1154-s001.docx]

**Supplementary Figures**


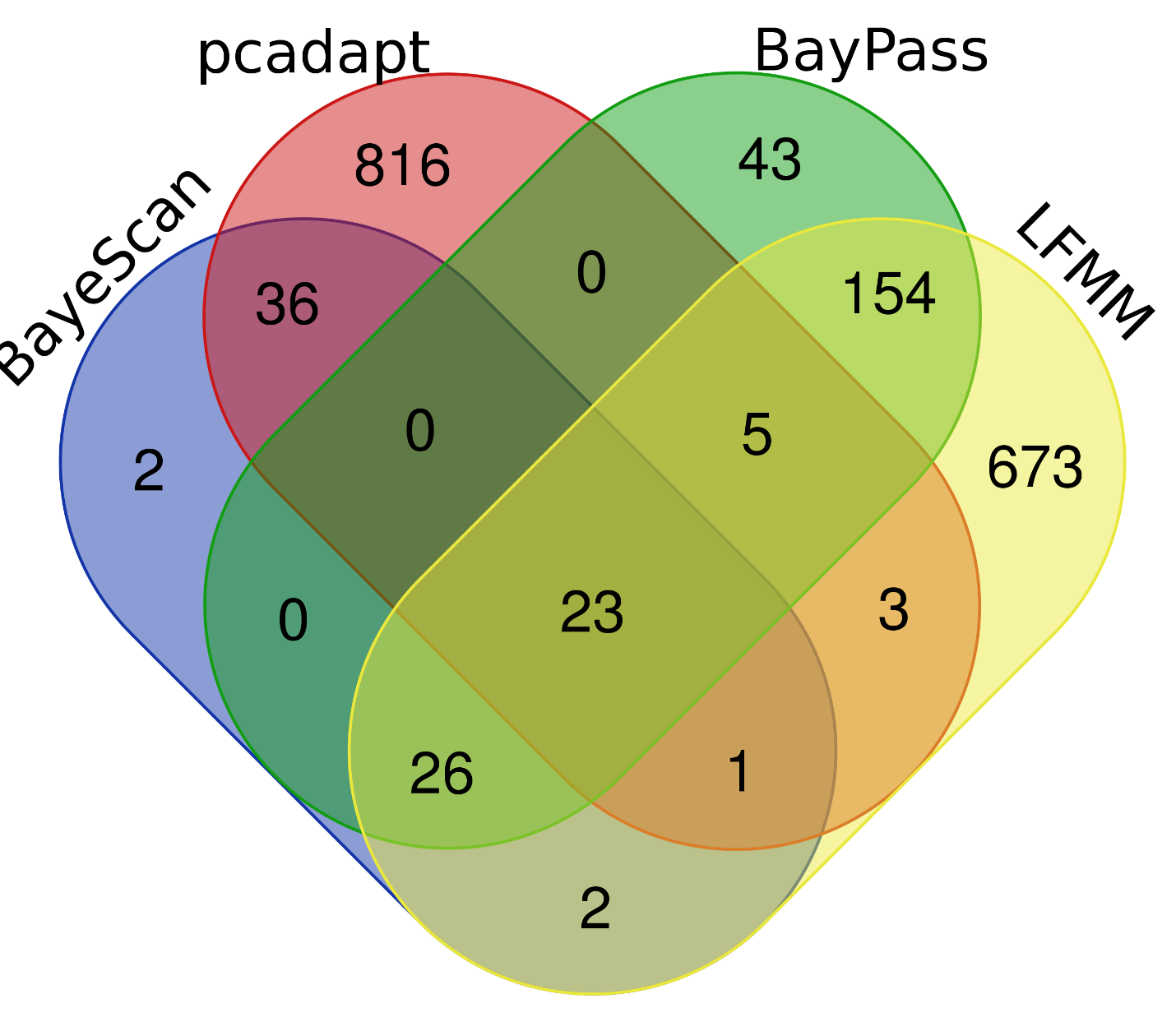


**Figure S1.** Venn Diagram showing overlapping outlier SNPs between BayeScan (Foll & Gaggiotti, 2008), pcadapt (Luu, Bazin, & Blum, 2017), BayPass (Gautier, 2015), and LFMM (Frichot & François, 2015). Diagram produced using http://bioinformatics.psb.ugent.be/webtools/Venn/. Results verified using R packages gplots and tidyverse.


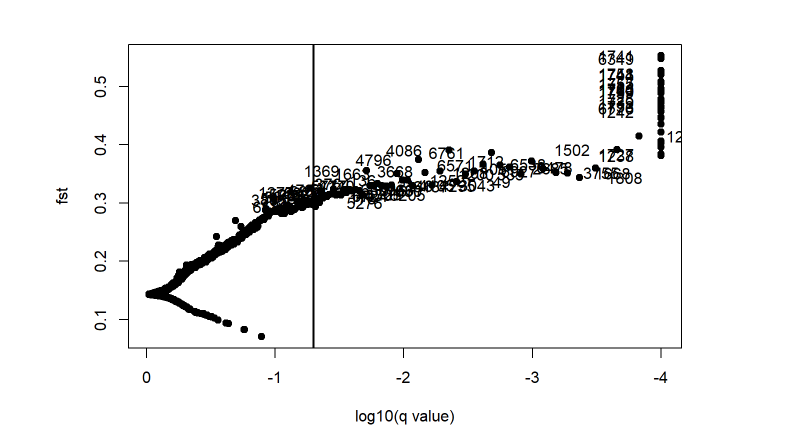


**Figure S2.** Results of the outlier detection using BayeScan (Foll & Gaggiotti, 2008). Vertical line corresponds to the q-value of 0.05.


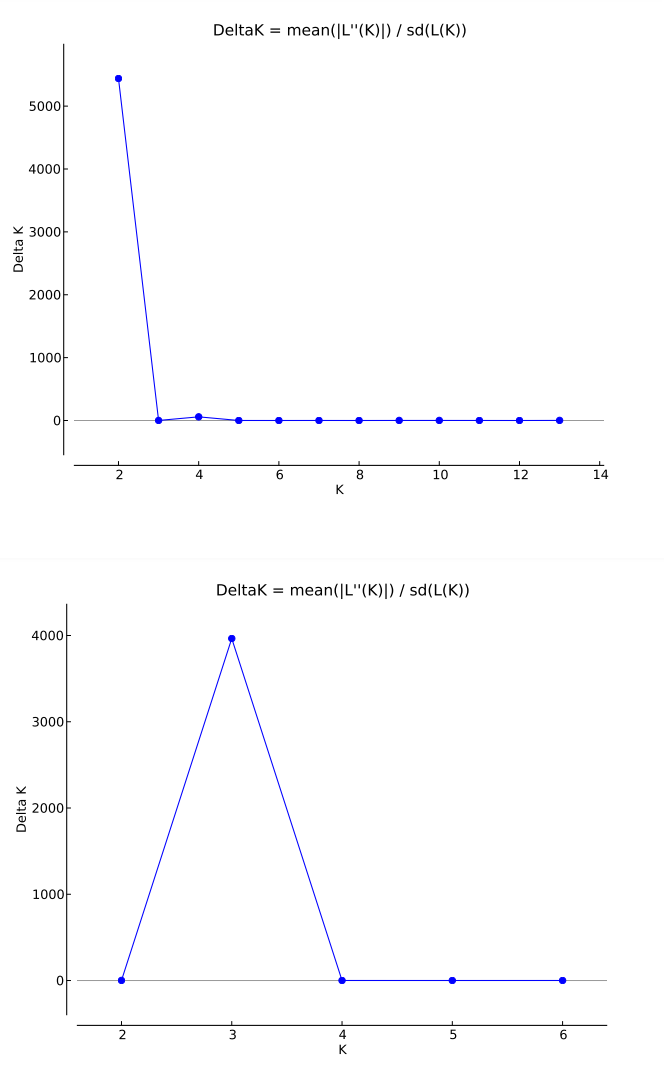


**Figure S3.** Results of the EVANNO analysis to determine the optimal number of clusters (Evanno et al., 2005), as implemented in STRUCTURE HARVESTER (Earl & vonHoldt, 2012). Top panel shows results for runs conducted using neutral SNPs; bottom panel shows results for runs conducted using robust outlier SNPs.
